# Integration of transcriptomics and metabolomics for understanding the global responses to neutral pH and high pH under high light in photosynthetic bacterium *Rhodobacter alkalitolerans* strain JA916^T^

**DOI:** 10.1128/aem.00932-25

**Published:** 2025-08-29

**Authors:** Mohammad Yusuf Zamal, Sureshbabu Marriboina, Vijay Srinivas Lavudiya, Jerome Xavier Gunasekaran, Aprajita Kumari, Kapuganti Jagadish Gupta, Venkata Ramana Chintalapati, Rajagopal Subramanyam

**Affiliations:** 1 Department of Plant Science, School of Life Sciences, University of Hyderabad, Gachibowli, Telangana, India; 2 National Center for Plant Genome Research, Aruna Asaf Ali Marg, New Delhi, Delhi, India; Washington University in St. Louis, St. Louis, Missouri, USA

**Keywords:** *Rhodobacter alkalitolerans *strain JA916^T^, high light, high pH, metabolome, transcriptome, L-proline, putrescine

## Abstract

Photosynthetic microorganisms are often exposed to fluctuating light intensities in their ecological niches. *Rhodobacter alklitolerans* is a versatile photosynthetic bacterium able to grow in alkaline conditions. Comprehensive analysis of transcriptomic and metabolomic data reveals preferential gene expression and metabolic regulation of *R. alklitolerans* strain JA916^T^ to alkaline pH (hpH) to neutral pH (npH) growth conditions under high light intensities. The transcriptome analysis provided that the majority of the transcripts related to photosystems were found to increase their expression in both npH and hpH under high-light conditions compared to low light conditions. Interestingly, reaction center transcripts (*pufH*, *pufM*, and *pufL*) and light-harvesting complex transcripts (*pufA* and *pufB*, *pucA* and *pucB*) were upregulated one- to two fold more in npH with increasing light intensity conditions. However, bacteriochlorophyll (BChl) *a* and carotenoid biosynthesis genes were significantly upregulated in hpH. We also found higher expression of cell division transcripts in npH conditions (*murG*, *ftsZ*, *ftsQ*, and *ftsA* with 1- to 1.5-fold) than in hpH. Furthermore, the metabolome analysis showed a higher correlation among the physiologically important metabolites such as putrescine, phenylalanine, phytol, L-lysine, L-proline, β-alanine, citric acid, L—5 oxoproline, and Pentanedioic acid. These were found to be part of several important metabolic pathways, including porphyrin metabolism, responsible for BChl metabolism, amino acid metabolism, and the tricarboxylic acid (TCA) cycle, playing a crucial role in energy production, in osmotic balance, and maintaining redox balance caused by high light. The real-time PCR analysis revealed that TCA cycle pathway genes also showed higher expression levels in npH than in hpH conditions under high light. Overall, the current study identifies candidate genes, metabolites, and alkaline-dependent high-light stress defense mechanisms that could potentially enhance alkali and high-light resilience in *R. alklitolerans* strain JA916^T^.

**IMPORTANCE:**

The study provides detailed information about the differential transcriptomic and metabolic response of the photosynthetic bacterium *R. alkalitolerans* strain JA916^T^ in alkaline and neutral pH conditions. The study puts emphasis on the core photosynthetic transcripts and how their expression level is lower in hpH compared to npH, especially when it is grown in high light. The study also reasons out the relatively higher expression of bacteriochlorophyll *a* and carotenoid biosynthesis pathway transcripts in hpH, emphasizing the more photoprotection in hpH growth conditions. Furthermore, it also explores the role of metabolites in the study under high light and pH conditions, such as L-proline, putrescine, phenylalanine, etc., and their intricate metabolic networks through correlation matrix and interaction networks. The high-light and alkali stress markers identified in our study from the photosynthetic bacterium *R. alkalitolerans* strain JA916T can be used to develop stress-tolerant strains.

## INTRODUCTION

Photosynthetic purple non-sulfur bacteria Rhodobacter show a great extent of metabolic and energetic diversity (1). One of the species of purple non-sulfur bacterium, *Rhodobacter (R.) sphaeroides*, is among the most extensively researched photosynthetic organisms. It has been utilized to create models that explore photon capture, light-driven energy metabolism, and various other facets of its diverse lifestyles (2, 3). Purple non-sulfur bacteria have also been investigated for their potential biotechnological applications, such as hydrogen production and the synthesis of ubiquinone and polyhydroxybutyrate. These compounds may serve as sources for biodegradable plastics, aid in the remediation of radioactive contamination, and contribute to the fixation of carbon dioxide and nitrogen (4, 5).

A metabolic study not only provides the clues to produce some biologically important molecules, such as ubiquinone and bacteriochlorophyll (BChl), in the case of photosynthetic purple non-sulfur bacteria. Also, different kinds of metabolites are produced in a given specific growth condition, such as osmolarity, salinity, and high light, signifying their important biological role in cell metabolism. The metabolic profile of astaxanthin accumulation in *Haematococcus pulvalis* in high-light conditions (6). In this study, they also found several other metabolites of different groups of amino acids, sugar alcohols, amines, nucleic acids, and a few others. The intermediates of the Calvin cycle and the Tricarboxylic acid (TCA) cycle serve as the precursors for these metabolites, suggesting that these two cycles contribute significantly to the production of cytoprotective metabolites. Similarly, adoptive strategies by transcriptomic and proteomic comparative study were reported in α-proteobacteria in photooxidative conditions (7). They found that sigma factors RpoE and RpoH_II_ are induced in both species. They also found the crtIB-tspO operon, which encodes proteins involved in the biosynthesis of carotenoid precursors and a regulator of photosynthesis, along with *cbiX*, which encodes a putative ferrochelatase, is activated in *R. capsulatus*.

*R. alklitolerans* strain JA916^T^ is one of the purple non-sulfur bacteria that can also grow in alkaline conditions. In this study, we have tried to understand the transcriptomic and metabolomic responses of *R. alklitolerans* in alkaline pH (npH) and neutral pH (hpH) along with different light intensities of 30, 250, and 500 µmol photons m^−2^s^−1^ in special relation to photosynthesis to find out which pathways and metabolites play an important role in the given condition. However, several studies have been made on the role of different metabolites in high light and different stresses like salinity and drought. Another study reported that the role of amino acid proline in several stresses, such as salinity, drought, heat stress, and heavy metal stress, also observed some polyols, such as phytol. The chlorophyll metabolism in Arabidopsis was found to involve phytol, which is one of the integral key molecules for chlorophyll degradation (8). Similarly, they studied the role of amine putrescine in salt-stressed cucumber seedlings and found that putrescine has the potential to enhance chlorophyll metabolism and the xanthophyll cycle by modulating enzyme activities and mRNA transcription levels, thereby increasing the salt tolerance of cucumber plants (9).

Growth of *R. alkalitolerans* was already established and standardized in our recent report (10). It was found that generation time decreased with an increase in light intensity more in npH than in hpH. Oxidative stress level was found to be higher in npH than in hpH with an increase in light intensity (10). In this study, the transcripts of photosynthetic apparatus, light-harvesting antenna, BChl *a*, and carotenoid biosynthesis pathways were analyzed, along with the peculiar and physiologically important metabolites, and their pathways of involvement were also analyzed.

## RESULTS

### Read alignment, principal component analysis, correlation matrix, and differential expression statistics analysis of the transcripts

Transcripts of *R. alklitolerans* were aligned against the phylogenetically closely related species *Rhodobacter azotoformans*, *R. sphaeroides*, and *Blastochloris viridis*. We found that transcripts of *R. alklitolerans* showed maximum mapped reads similarity with *R. azotoformans* (47.28%), *R. sphaeroides* (43.4%), and *B. viridis* (33.57%; Ensembl bacteria database; see Fig. S1A at https://github.com/2902-myz/Supplementary-Data-). The principal component analysis (PCA) showed a strong variation in the transcripts of 30 µmol photons m^−2^s^−1^ npH and 500 µmol photons m^−2^s^−1^ npH, with PC1 88.97%, whereas a sample of 30 hpH vs 500 hpH, with PC2 8.02% indicates lesser variation because of high light (see Fig. S1B at https://github.com/2902-myz/Supplementary-Data-). The hierarchical correlation clustering analysis showed a high correlation between and within the replicates in both npH and hpH conditions, and correlation values ranged from 0.96 to 0.99 (see Fig. S1C at https://github.com/2902-myz/Supplementary-Data-). To find out the expression levels of differentially expressed transcripts, our data were categorized into four groups (i) 30 npH vs 30 hpH, (ii) 30 npH vs 500 npH, (iii) 30 hpH vs 500 hpH, and (iv) 500 npH vs 500 hpH, and the data were represented with a heat map by calculating a *z*-score for each row from the normalized read counts of the samples (see Fig. S2 at https://github.com/2902-myz/Supplementary-Data-). A total of 2,309 differentially expressed genes were identified. To simplify our data, the total differentially expressed transcripts were portioned and represented as comparative groups: a total of 1,279 transcripts in 30 npH vs 500 npH, about 596 transcripts in 30 hpH vs 500 hpH, 877 transcripts in 30 npH vs 30 hpH, and 539 transcripts in 500 npH vs 500 hpH. Furthermore, the differentially upregulated and downregulated genes were segregated (see Fig. S1D at https://github.com/2902-myz/Supplementary-Data-).

**Fig 1:**
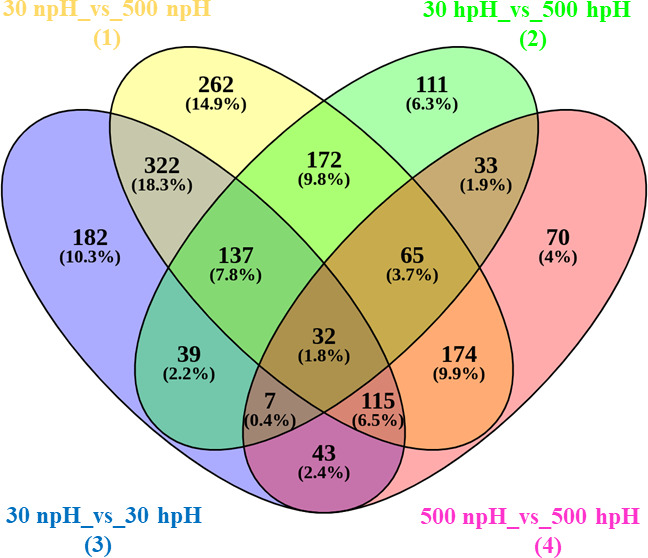
Venn diagram showing the differentially expressed genes in particular and in common genes in comparisons of 30 npH vs 500 npH, 30 hpH vs 500 hpH, 30 npH vs 30 hpH, and 500 npH vs 500 hpH.

A Venn diagram was used to find out the common and exclusively expressed genes in each comparative condition (Fig. 1). Four comparative groups were made to show the expression levels of transcripts between the samples (i) 30 npH vs 500 npH, (ii) 30 hpH vs 500 hpH, (iii) 30 npH vs 30 hpH, and (iv) 500 npH vs 500 hpH. Overall, a total of 1,279 significantly differentially expressed transcripts were detected in 30 npH vs 500 npH, out of which 262 genes were exclusively expressed in this comparative group. The rest of the transcripts were shared by all different comparative groups: 322 transcripts in 30 npH vs 30 hpH, 174 transcripts in 500 npH vs 500 hpH, and 172 transcripts in 30 hpH vs 500 hpH. Likewise, 182 genes were exclusively expressed in 30 npH vs 30 hpH, 70 transcripts in 500 npH vs 500 hpH, and 111 transcripts in 30 hpH vs 500 hpH. There were 137 transcripts commonly expressed in 1, 2, and 3 groups and 65 transcripts in 1, 2, and 4 groups. Similarly, there were 32 transcripts, which were expressed commonly in all the conditions (Fig. 1). These genes are majorly related to porphyrin synthesis (*bchN*, *bchX*, *hemN_1*, *bchF*, and *bchY*), amino acid (*hemA*, *acdA*, and *ODC1*), carbohydrate (*G6PD*, *fdhF*, and *fdoG*), and nucleotide metabolism (nrdA).

The differentially expressed genes in all four comparative groups were divided into significantly upregulated and downregulated genes and were collectively represented in a volcano plot. In the comparative pair of 30 npH vs 30 hpH, the upregulated genes included protein-coding genes, *ZoOX0XrerWWHp4e*, *JS-Tis32Pk-Mgdl*, *X8A8470fzF7V1nD*, which are the bifunctional coenzyme of pyrroloquinoline quinone (PQQ) synthesis protein, PqqA peptide cyclase, and hydrazine synthase subunit β, respectively. Whereas the downregulated genes, namely *Zzpq7XXteZDfG72*, *BH65nYbTgCscS_h*, *w24Ijsh3eHFHcek*, and *cshYMyQWrrvbMGM*, which are formyl tetrahydrofolate deformylase, NADP-reducing hydrogenase subunit HndC, these two are involved in carbohydrate metabolism, specifically in glyoxylate and dicarboxylate metabolism. Furthermore, methyl malonyl-CoA carboxyltransferase 12S subunit is involved in amino acid metabolism, specifically, in leucine, isoleucine, and valine metabolism, and the last one is 10 kDa chaperonin 1, respectively. The genes in 30 npH vs 500 npH group included gene *O09RxdHEd-hx4jR*, a proprotein coding gene playing a role in porphyrin metabolism; *UmU8DD29LcOhjzL*, which is a light-independent protochlorophyllide subunit L and is also involved in porphyrin metabolism; and *Q5Q9ffm8pa5v03N* (*fusa1*) is an elongation factor G (see Fig. S3B at https://github.com/2902-myz/Supplementary-Data-).

High fold upregulated genes in 30 hpH vs 500 hpH included gene *irtRfPpZQftHVYz* (*yvyD*) taking part in ribosome hibernation promotion factor, gene *rLnKPvHGs2pWyEY* (*ptsN*) involved in environmental information processing by taking part in phosphotransferase system, gene *Ayz9u_2-T8PPkUN* (*bchJ*) involved in porphyrin metabolism of bacteriochlorophyll biosynthesis, and gene *bpC4iz1H03QfDZ2* (*bchX*) also involved in porphyrin metabolism and acting as chlorophyllide reductase (see Fig. S3C at https://github.com/2902-myz/Supplementary-Data-).

The downregulated genes included *33CZuO0ewjmmGnV* (*rhlE_1*), which is an ATP-dependent RNA helicase involved in folding, sorting, and RNA degradation; *ZVGJct6QohSuPuT* (*rpsG*), a 30S ribosomal protein S7 involved in genetic information processing; *1H914DAS46GtfDp* (*rpsQ*) involved in 30S ribosomal protein S17, also involved in genetic information processing at ribosome level; and *J6LHMfcNPYJWrwu* (*adk_2*), an adenylate kinase involved in nucleotide metabolism of purine and thiamine (see Fig. S3C at https://github.com/2902-myz/Supplementary-Data-). Likewise, in the comparative pair of 500 npH vs 500 hpH, the highly upregulated and downregulated genes included *3k0iepaggW2qjap* (*futA1*), which encodes the iron uptake protein A1, and *0Excr0RlLe_gM6k* (*ybhL_2*), which encodes the inner membrane protein *YbhL* (see Fig. S3D at https://github.com/2902-myz/Supplementary-Data-).

### Comparative transcript-level expression of reaction center and light-harvesting genes

**Fig 2:**
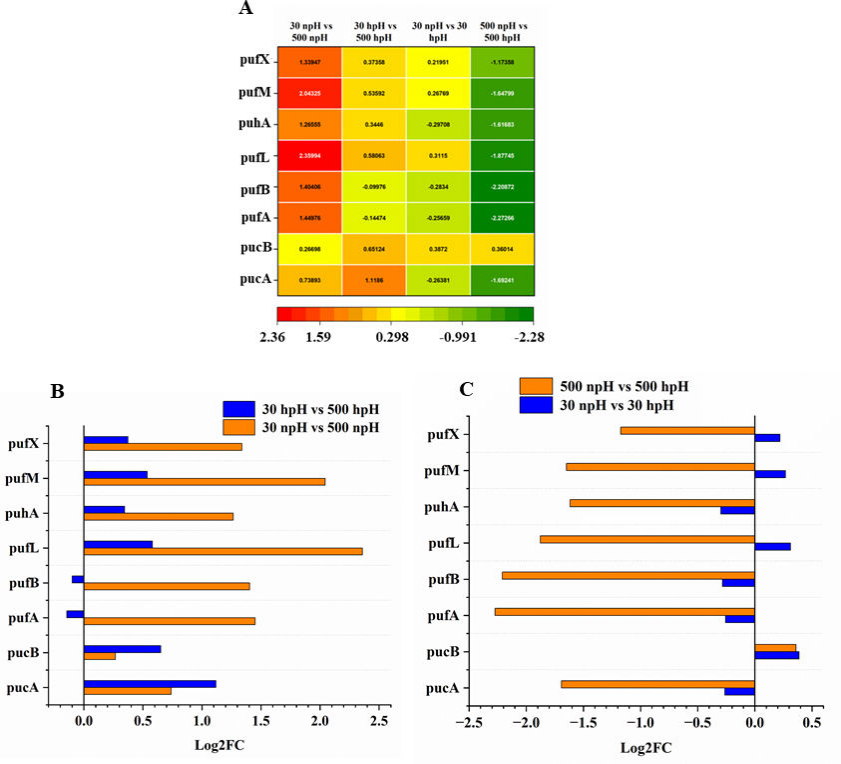
Fold change expression analysis of the transcripts of photosystem reaction center and antenna transcripts in comparative form of 30 µmol photons m^–2^s^–1^ npH vs 30 µmol photons m^–2^s^–1^ hpH, 30 µmol photons m^–2^s^–1^ npH vs 500 µmol photons m^–2^s^–1^ npH, 30 µmol photons m^–2^s^–1^ hpH vs 500 µmol photons m^–2^s^–1^ hpH, and 500 µmol photons m^–2^s^–1^ npH vs 500 µmol photons m^–2^s^–1^ hpH. (**A**) Heatmap representation of the fold change expression of the transcripts. (B and C) Bar graph comparison between same light intensities in different pH conditions.

To find out the expression levels of core photosystem reaction center (RC) transcripts and light-harvesting antenna transcripts, the data were represented separately as a heatmap and bar plots. The overall expression level of all the RC transcripts and antenna transcripts was presented in the heatmap (Fig. 2A). Reaction center transcripts such as *pufM* (M), *pufL* (L), *puhA* (H), as well as light-harvesting antenna transcripts *pucA* (α) and *pucB* (β), which code for LH2 α and β, and *pufA* and *pufB*, which code for LH1 α and β, were upregulated with increased light intensity.

The fold change expression was more in npH condition comparisons, i.e., 30 npH vs 500 npH (>2.5-fold) than that of the 30 hpH vs 500 hpH (>1.1-fold). The LH1 transcripts were slightly downregulated in 30 hpH vs 500 hpH (Fig. 2B). Furthermore, the comparison between 30 npH vs 30 hpH and 500 npH vs 500 hpH groups confirmed most of the RC and antenna transcripts were downregulated in hpH conditions than npH (Fig. 2C). Transcript expression of transmembrane protein PufX, which plays an important role in RC-LH1 dimer formation, was also upregulated in the npH group compared to the hpH group (Fig. 2B). Figure 2C showed *pufX* transcript expression levels were lower in the 500 hpH condition than in 500 npH, whereas they were lesser in 30 npH than 30 hpH.

**Fig 3:**
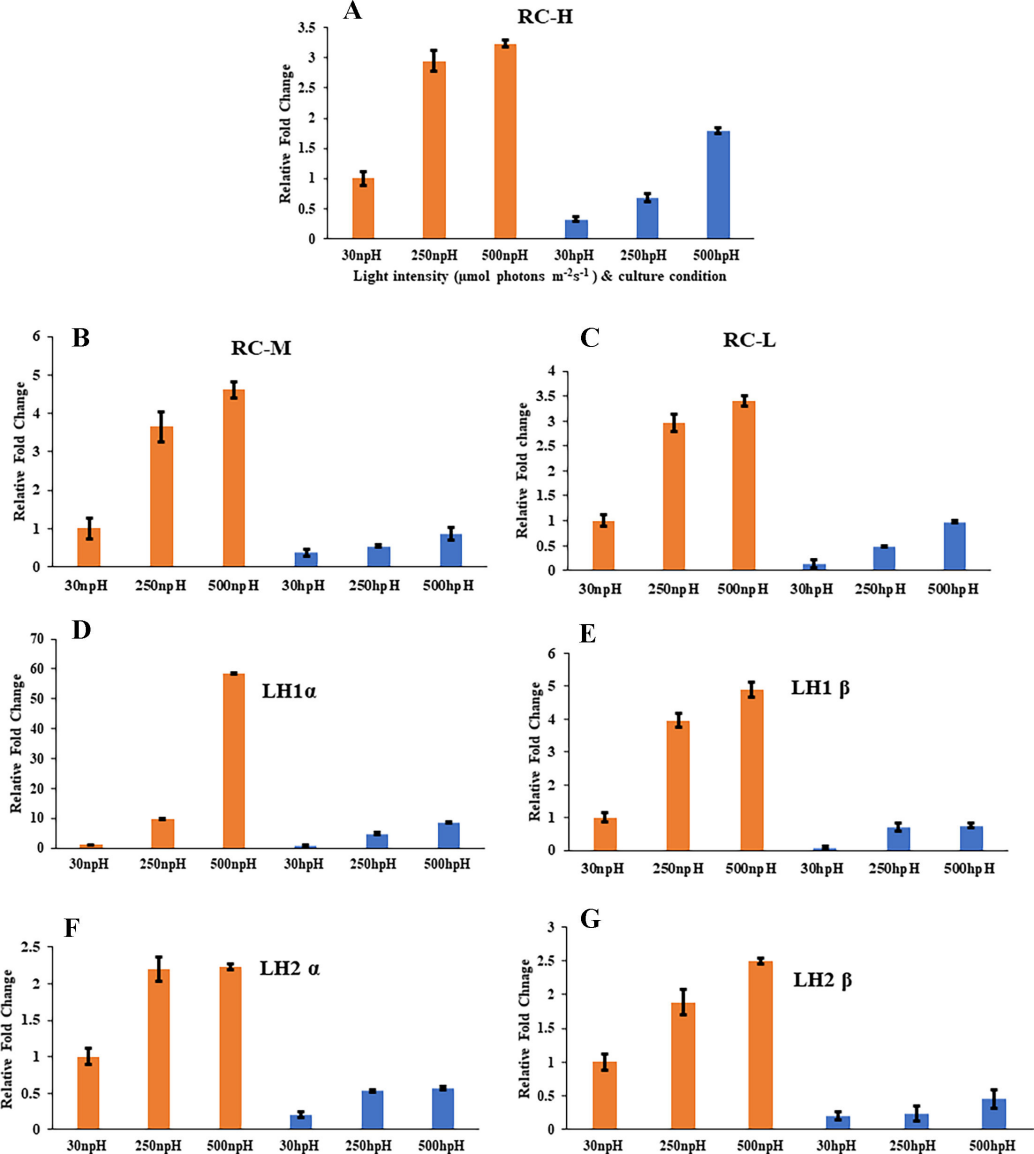
qRT-PCR expression analysis of the reaction center transcripts (A) RC-H (*puhA*), (B) RC-M (*pufM*), and (C) RC-L (*pufL*), as well as (D and E) LH1 antenna transcripts LH1 α and β (*pufA* and *pufB*) and (F and G) LH2 α and β (*pucA* and *pucB*).

Furthermore, quantitative real-time PCR (qRT-PCR) analysis of reaction center proteins showed that the expression level of transcripts is higher in npH conditions than that of the hpH conditions, and it has increased with the increasing light intensity (Fig. 3). All the reaction center and light-harvesting antenna transcripts were relatively less abundantly expressed in hpH than npH conditions.

### Transcript expression levels of bacteriochlorophyll *a* biosynthesis pathway genes and their RT-PCR validation

Bacteriochlorophyll *a* is the major light-harvesting pigment in the photosystem of *R. alkalitolerans*. It makes the dimer of a special pair of bacterial reaction centers. BChl *a* biosynthesis includes a cascade of enzymatic conversions involving the *chlL*, *chlN*, *bchY*, *bchZ*, *bchF*, *bchC*, *bchG*, and final *chlP* catalyzing conversion of geranylgeranyl bacteriochlorophylloide *a* to BChl *a*.

**Fig 4:**
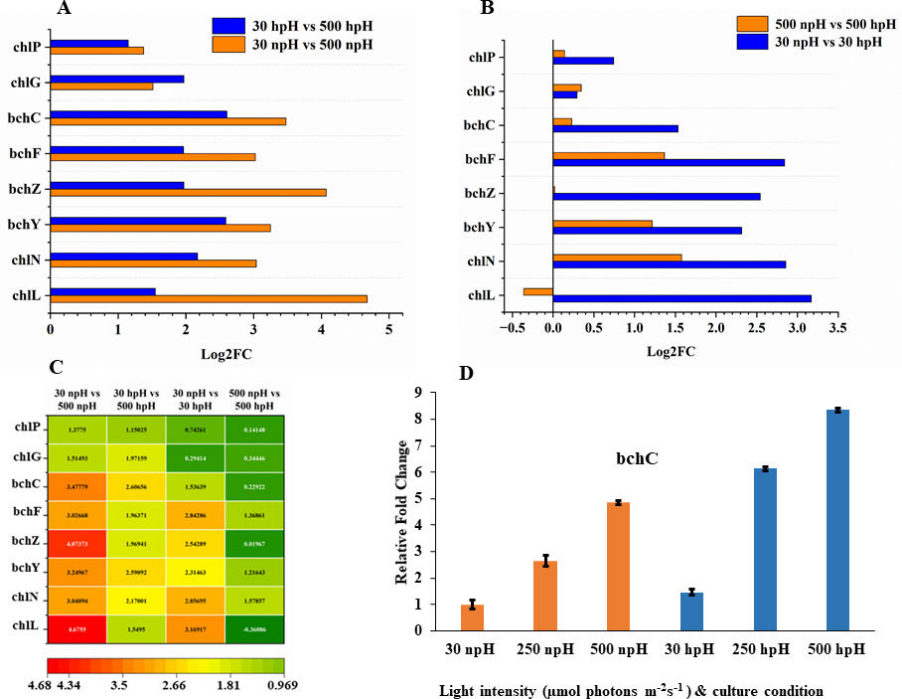
Transcript expression level of photosynthetic BChl *a* bisynthesis pathway genes. (A) Relative fold change expression analysis in comparisons between 30 µmol photons m^−2^s^−1^ npH vs 500 µmol photons m^−2^s^−1^ npH, 30 µmol photons m^−2^s^−1^ hpH vs 500 µmol photons m^−2^s^−1^ hpH, (B) 30 µmol photons m^−2^s^−1^ npH vs 30 µmol photons m^−2^s^−1^ hpH, and 500 µmol photons m^−2^s^−1^ npH vs 500 µmol photons m^−2^s^−1^ hpH. (**C**) Heatmap representation of the fold change expression of the transcripts. (**D**) Relative qRT-PCR expression of BChl *a* synthesizing gene *bchC*.

The fold change expression from RNA-seq data shows higher expression in npH conditions than hpH in comparative pairs of 30 npH vs 500 npH and 30 hpH vs 500 hpH, and fold change is relatively higher in npH conditions (Fig. 4A). However, the inter-comparative pairs of 500 npH vs 500 hpH and 30 npH ns 30 hpH show that the expression level of transcripts is higher in hpH than in npH conditions (Fig. 4B). The same expression levels are also presented in heatmap analysis (Fig. 4C). The qRT-PCR validation of the *bchC* gene confirms the expression level to be higher in hpH conditions than npH conditions.

### Transcript expression level of the carotenoid biosynthesis pathway gene and its RT-PCR validation

The only carotenoid found in *R. alkalitolerans* is spheroidene, which is synthesized from geranylgeranyl diphosphate by genes involving *crtB*, *crtI*, crtC, *crtD*, and the final biosynthesis step, *crtF*, converting dimethylspheroidene to spheroidene. Gene *crtA* converts spheroidene to spheroidenone depending upon the redox status of the cell (11).

**Fig 5:**
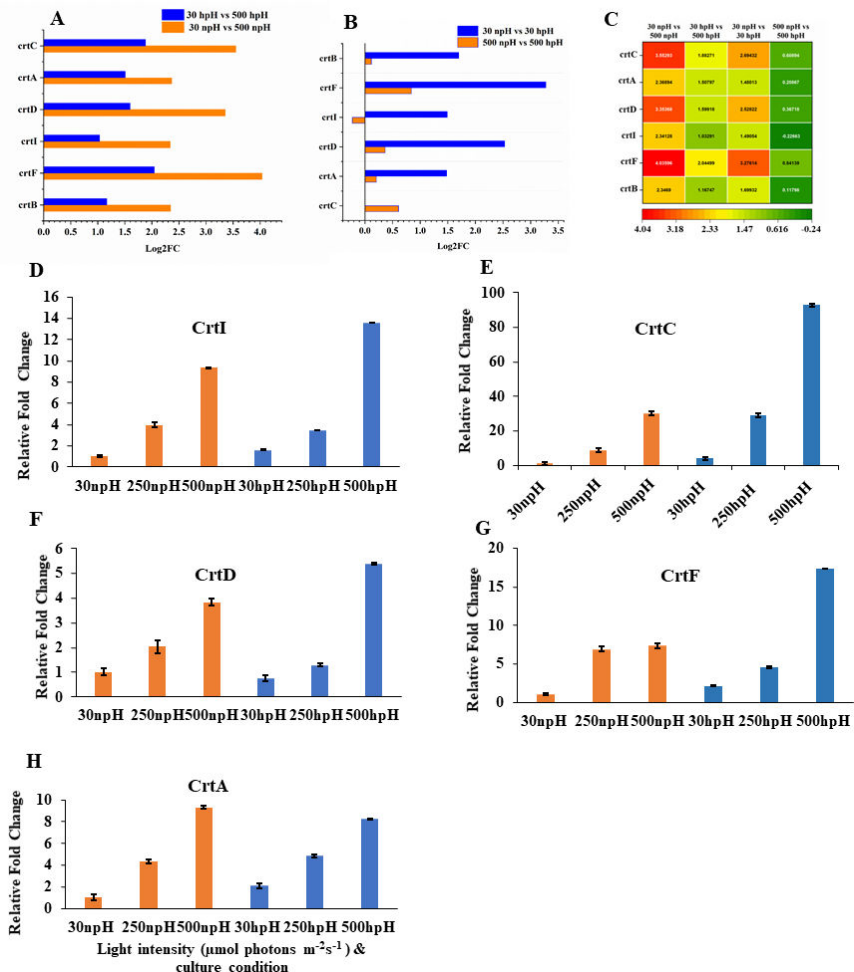
Transcript expression level of photosynthetic carotenoid sphaeroidene bisynthesis pathway genes. (A) Relative fold change expression analysis in comparisons between 30 µmol photons m^−2^s^−1^ npH vs 500 µmol photons m^−2^s^−1^ npH, 30 µmol photons m^−2^s^−1^ hpH vs 500 µmol photons m^−2^s^−1^ hpH, (B) 30 µmol photons m^−2^s^−1^ npH vs 30 µmol photons m^−2^s^−1^ hpH, and 500 µmol photons m^−2^s^−1^ npH vs 500 µmol photons m^−2^s^−1^ hpH. (**C**) Heatmap representation of the fold change expression of the transcripts. (**D−H**) Relative qRT-PCR expression of BChl *a* synthesizing genes *crtI*, *crtC*, *crtD*, *crtF*, and *crtA*.

These carotenoid biosynthesis pathway genes were also increased with an increase in light intensity. The fold expression was higher in npH; however, the overall expression level was higher (0.5- to 3.5-fold) in hpH conditions and is evident from the inter-comparative fold change expression data (Fig. 5B). Furthermore, the qRT-PCR analysis of the genes also showed higher expression in hpH than npH conditions (Fig. 5D to G). Another gene, *crtA*, which converts spheroidene to spheroidenone, was also expressed and was relatively higher in 30 hpH condition; however, at 500 µmol photons m^−2^s^−1^, the expression is higher in npH than in hpH condition (Fig. 5H).

### Transcript expression level of cell division genes and RT-PCR validation of a few transcripts and principal component analysis

**Fig 6:**
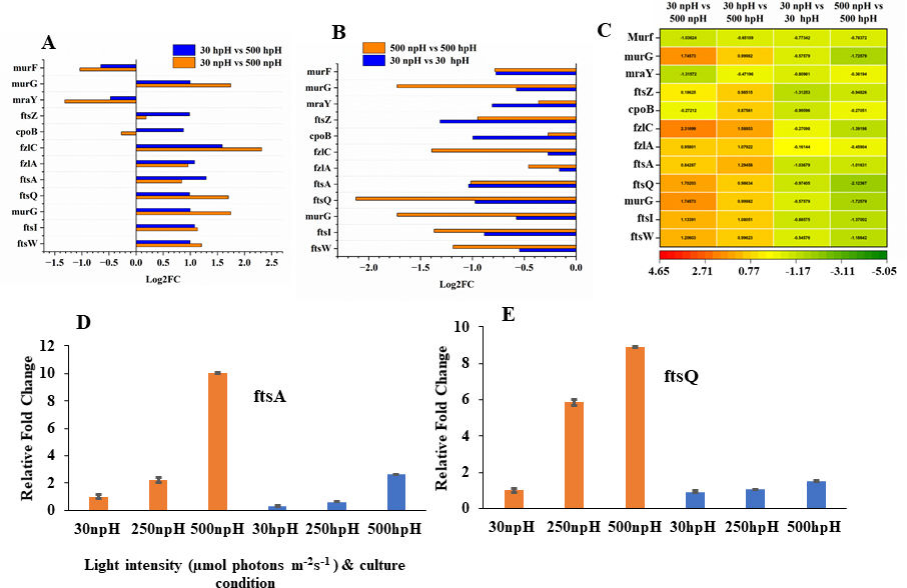
Fold change expression of cell division transcripts obtained from transcriptomic data. (**A**) Fold change expression showing comparative view of 30 µmol photons m^−2^s^−1^ npH vs 500 µmol photons m^−2^s^−1^ npH and 30 µmol photons m^−2^s^−1^ hpH vs 500 µmol photons m^−2^s^−1^ hpH. (**B**) 30 µmol photons m^−2^s^−1^ npH vs 30 µmol photons m^−2^s^−1^ hpH and 500 µmol photons m^−2^s^−1^ npH vs 500 µmol photons m^−2^s^−1^ hpH. (**C**) Heat map representation of the fold change expression. Relative qRT-PCR expression of cell division transcripts of *ftsA* (D) and *ftsQ* (E).

As explained and reported earlier (10), the generation time has decreased with increasing light intensity, and the generation time is relatively lesser in npH than in hpH. A heatmap was constructed to find out the expression levels of genes involved in cell division and their fold change in the comparative pairs: 30 npH vs 500 npH, 30 hpH vs 500 hpH (Fig. 6A), 500 npH vs 500 hpH, and 30 npH vs 30 hpH (Fig. 6A through C). The majority of the genes involved in cell division, such as *murG*, *ftsZ*, *cpoB*, *fzlC*, *fzlA*, *ftsA*, *ftsQ*, *ftsI*, and *ftsW*, were upregulated in high-light conditions, and their expression level was higher in npH conditions. However, the expression of transcripts *murF* and *mraY* was downregulated in both npH and hpH conditions in high light (Fig. 6A).

Furthermore, the qRT-PCR validation of genes *ftsA* and *ftsQ* was also highly upregulated in npH conditions in all three light intensities of 30, 250, and 500 µmol photons m^−2^s^−1^ compared to hpH conditions. Although the expression in hpH conditions is relatively lesser, in hpH also, the expression is upregulated with an increase in light intensity (Fig. 6D and E).

Metabolite groups obtained from PCA showed that the variation between the metabolites in the npH category was not very different in comparative pairs of 30 npH vs 250 npH and 30 npH vs 500 npH. It showed 75.6% and 75% variation, respectively (see Fig. S4A and B at https://github.com/2902-myz/Supplementary-Data-). However, the variation between the comparative data set of hpH was more than npH. It was found to be 84.4% between 30 hpH vs 250 hpH and 94.5% between 30 hpH vs 500 hpH (see Fig. S4C and D at https://github.com/2902-myz/Supplementary-Data-).

### Correlation-based clustering of the metabolites in *R. alkalitolerans*

**Fig 7:**
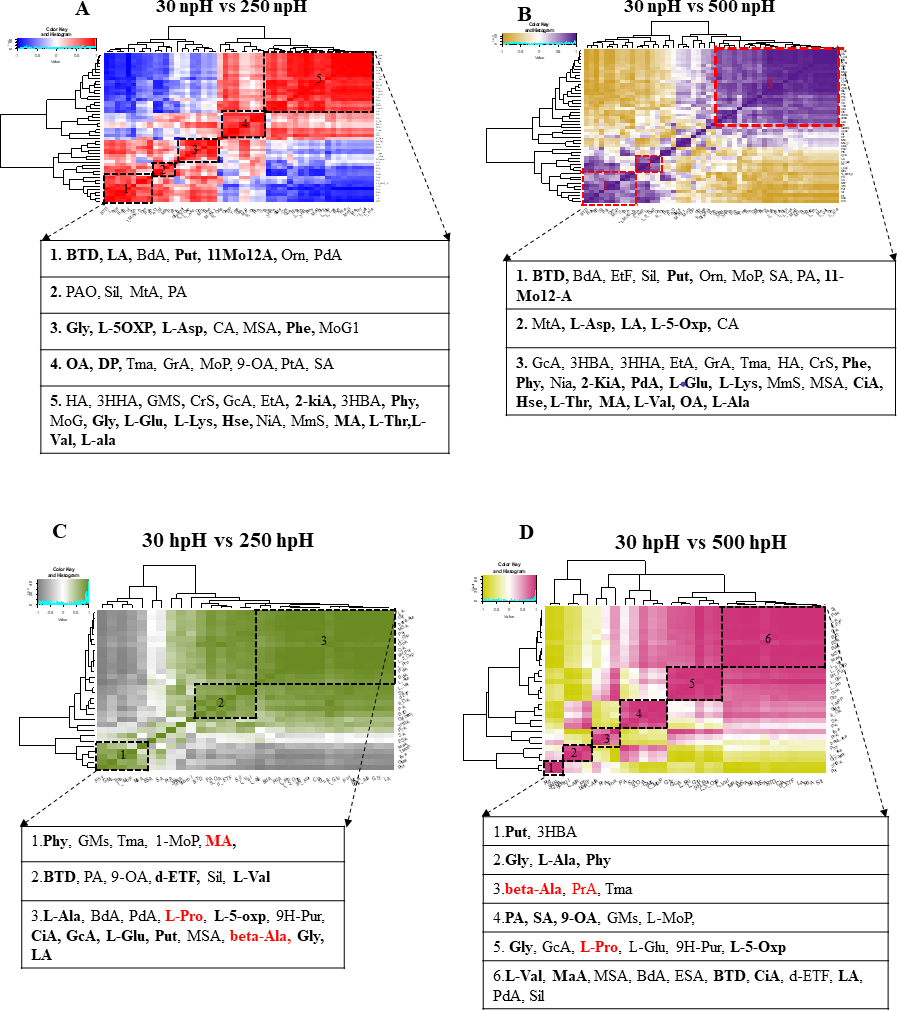
Correlation heat map and hierarchical clustering of metabolites. (**A**) 30 npH vs 250 npH, (**B**) 30 npH vs 500 npH, (**C**) 30 hpH vs 250 hpH, and (**D**) 30 hpH vs 500 hpH. Each correlation value (based on Pearson correlation coefficient) corresponds to an average of three biological replicates. The values 30 and 500 represent the culture growth light intensity in µmol photons m^−2^s^−1^.

To find out the correlation among the metabolites of different comparative groups of npH and hpH, the hierarchical correlation clustering analysis (HAC) was performed based on the Carl Pearson value (Fig. 7). HAC was performed on the individual fold values of metabolites obtained from each experimental condition: npH (50 metabolites) and hpH (30 metabolites). The metabolite correlations were analyzed using comparative group pairs as follows: 30 npH vs 250 npH and 30 npH vs 500 hpH (Fig7A and B).

The metabolites were strongly correlated, as given in Table S1 (https://github.com/2902-myz/Supplementary-Data-). In the comparative group, 30 npH vs 250 npH and 30 npH vs 500 npH metabolites were correlated and grouped depending on their correlation intensity. Fig. 7A and B showed that the metabolites in the first square belonged to different groups of metabolites, such as alcohols, carboxylic acids, diamines, and non-protein amino acids. Furthermore, the positively correlated metabolites were joined by some additional metabolites in the group of 30 npH vs 500 npH, such as Etf, Sil, Mop, Sta, and PA, which belong to sugar, alcohol, monoacylglycerol, fatty acid, and ions, respectively. Similarly, metabolites of a second group, including Pao, MtA, Sil, and Pra, were present in the group of 30 npH vs 250 npH. These metabolites belong to carboxylic acid, purines, alcohol, and ions, respectively. In the 30 npH vs 500 npH group, metabolites, namely, MtA, L-Asp, LA, L-5-Oxp, and CA, which are related to purine, amino acid, and amino carboxylic acid, correlated positively with each other (Fig. 7B). The metabolites in 30 npH vs 250 npH were given in two extra correlating groups, which included some important metabolites such as Gly, L-5Oxp, L-Asp, Phe, OA, and DP, which belong to amino acid, carboxylic acid, and ester. Moreover, the last group included some metabolites, mainly amino acids such as L-Glu, L-Lys, L-Thr, L-Val, and L-Ala, and some other metabolites such as MA, Hse, Gly, 2-KiA, and Phy, playing an important role in amino acid synthesis and photosynthesis. In contrast to it, the metabolites of 30 npH vs 500 npH group showed high interaction among each other than the rest of the metabolites present in the other groups, which is indicative of their importance in high-light condition and included some peculiar metabolites such as Phe, Phy, and CiA, apart from those found in 30 npH vs 500 npH (Fig. 7B). A total of 30 metabolites were recorded in the hpH group common to all replicates (see Table S1 at https://github.com/2902-myz/Supplementary-Data-); however, in hpH, it accumulated some metabolites that have been shown to play an important role in osmotic stress protection. The metabolites in the hpH group, in a comparative pair of 30 hpH vs 250 hpH, were converged into only three major correlating units, which included very important metabolites from different types of functional groups, some of which were also found in npH conditions (see Table S2 at https://github.com/2902-myz/Supplementary-Data-), as well as metabolites which were only detected in hpH, and these included L-Pro, β-Ala, and Pra.

### Network analysis of metabolites in npH and hpH under high-light conditions

**Fig 8:**
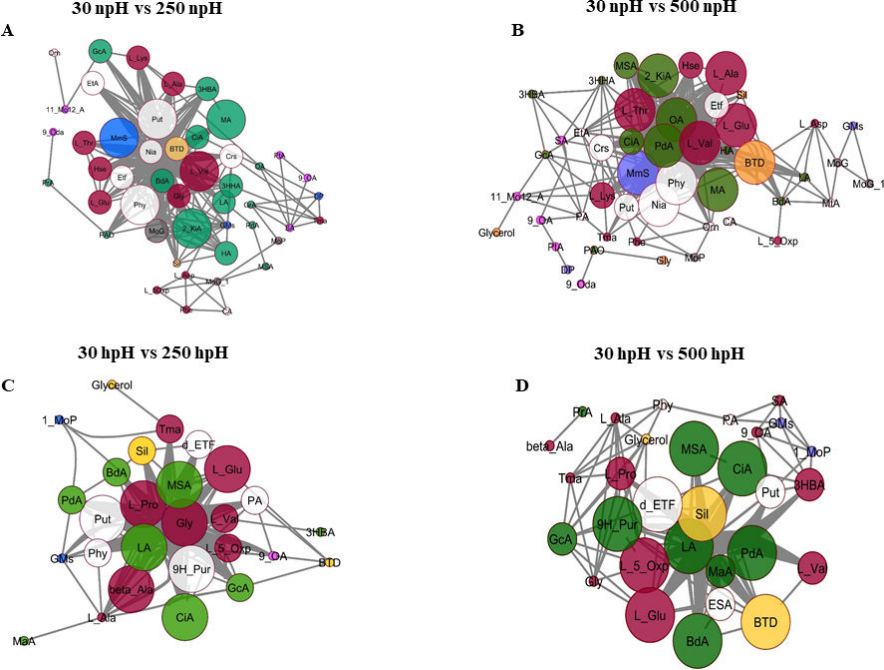
Correlation network of metabolites based on Pearson's coefficient with a probability threshold of *P* < 0.05. Metabolites are shown in colors and represented as nodes: yellow (alcohol metabolites), white (miscellaneous metabolites), red color (fatty acid metabolites), blue color (ester), orange color (nucleotide metabolism), and green color (carboxylic acid). Three different circle sizes change based on the degree of interaction; the bigger size circle represents having a greater number of interacting partners. The stroke represents interactions with other metabolites. (**A**) 30 npH vs 250 npH, (**B**) 30 npH vs 500 npH, (**C**) 30 hpH vs 250 hpH, and (**D**) 30 hpH vs 500 hpH represent the interaction among metabolites in each respective comparative groups.

To find out the interaction network of the metabolites, the Pearson correlation (*r*) was performed, and our major network plots were made depending upon the comparative pairs of 30 npH vs 250 npH (Fig. 8A) and 30 npH vs 500 npH (Fig. 8B). The metabolites were represented as nodes (round circles) and interacting with other neighboring metabolites. Interaction among the metabolites was made in four comparative groups, i.e., 30 npH vs 250 npH (Fig. 8A), 30 npH vs 500 npH (Fig. 8B), 30 hpH vs 250 hpH (Fig. 8C), and 30 hpH vs 500 hpH (Fig. 8D). The metabolites that had a greater number of interacting partners were higher. In comparative pairs of npH, the majority of the metabolites were found to interact with other metabolites, which include amino acids such as L-Lys, L-Ala, L-Thr, Hse, L-Glu, Gly, and L-Val. L-valine had major interacting patterns in 30 npH vs 250 npH group, increasing light intensity causing the interaction of these amino acid metabolites, which specifically included L-Ala, L-Thr, L-Val, and L-Glu, whereas Hse and L-Lys were reduced. Other metabolites such as Phy, MmS, MA, 2KiA, Put, and Btd were shown to have more interaction (Fig. 8A), and some of these metabolites, such as Phy, Nia, Btd, MA, MmS, OA, PdA, and 2-KiA, increased their interaction network in npH under high-light conditions. These metabolites belonged to various functional groups such as carboxylic acid, alcohol, amide, ester, etc.

The metabolites of hpH group include amino acids such as L-Glu, Gly, β-Ala, L-Val, L-5-Oxp, and L-Pro, which had bigger circles and had more interactions (Fig. 8C). It also included a few metabolites such as MSA, LA, CiA, GcA, PdA, Put, 9H-Purine, Put, etc, which represent the other metabolites from carboxylic acid, diamine, nucleotide, and sugar, with an increase in light intensity in 30 hpH vs 500 hpH; the amino acids L-5-oxp and L-Glu were found to have more interaction; however, the other amino acids had reduced interaction. However, the other metabolites such as Put, d-ETF, 9H-Pur, Phy, Sil, MSA, CiA, BTD, BdA, MA, CA, and LA were found in the interacting network, which represents their role in hpH under high-light conditions.

### Pathway enrichment analysis of metabolites

**Fig 9:**
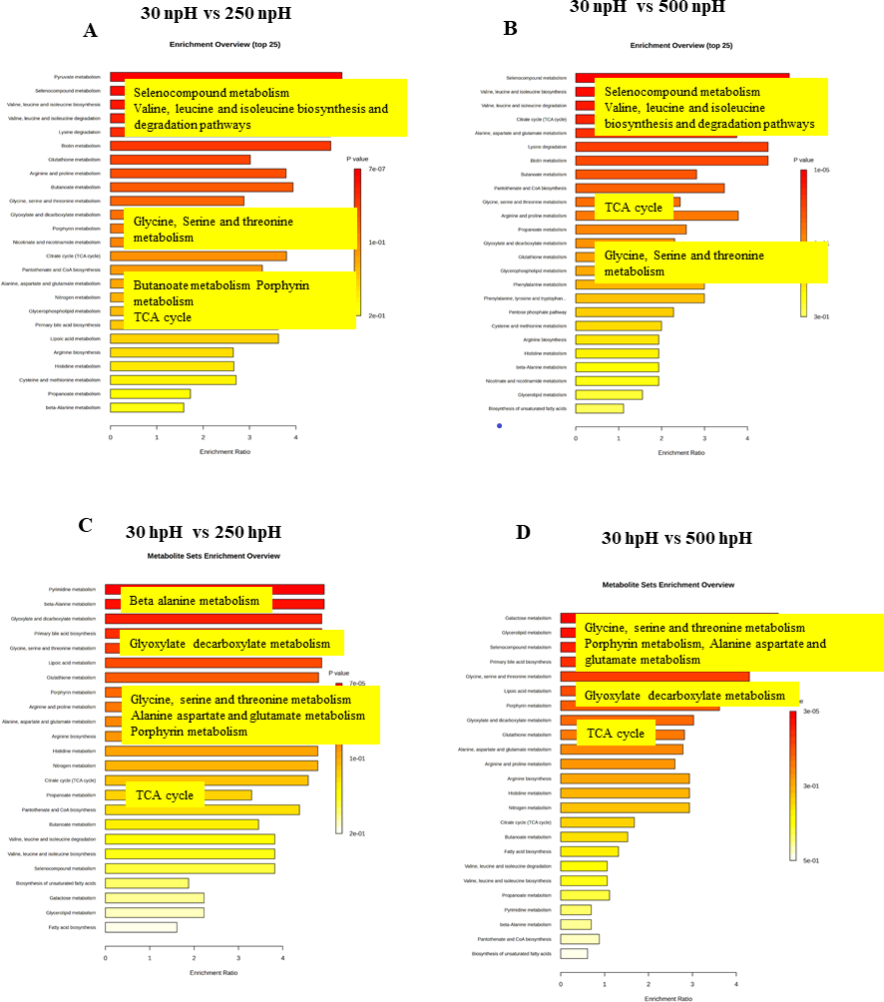
Analysis of metabolic pathways in npH and hpH culture conditions at light intensities of 30 and 500 µmol photons m^−2^s^−1^. Results were obtained from Metaboanalyst software version 6.0. Each bar represents a metabolic pathway with a *P* value < 0.05 to have significant changes. (A) 30 npH vs 250 npH, (B) 30 npH vs 500 npH, (C) 30 hpH vs 250 hpH, (D) 30 hpH vs 500 hpH. The values 30 and 500 represent the culture growth light intensity in µmol photons m^−2^s^−1^.

To find out the metabolic pathways related to the metabolites obtained in the analysis, Kyoto Encyclopedia of Genes and Genomes (KEGG) pathway enrichment analysis was performed in all four comparative pairs in npH and hpH. Fig. 9A and B represent the metabolic pathways enriched in npH high-light conditions compared to the control. These pathways were mostly from protein metabolism, which included seleno-compound metabolism, valine, leucine, isoleucine degradation and biosynthesis pathway, lysine degradation pathway, glutathione, arginine, and proline metabolism.

It also included glycine, serine, and threonine metabolism and citrate cycle (Fig. 9A). Similarly, in 30 npH vs 500 npH, the metabolically top-enriched pathways were almost the same, including seven compound metabolism; valine, leucine, and isoleucine degradation and biosynthesis pathway; citrate cycle; alanine aspartate, and glutamate metabolism; and glycine, serine, and threonine. In 30 npH vs 500 npH, the aromatic amino acid metabolism, named phenylalanine (Phe), tyrosine, and tryptophan metabolism, was also enriched (Fig. 9B). Similarly, the pathway enrichment analysis was also done for the hpH conditions to find out the enriched metabolic pathways in the comparative pairs of 30 hpH vs 250 hpH and 30 hpH vs 500 hpH. The enriched pathways included pyridine and β-alanine metabolism, which were absent in the npH conditions and glyoxylate and decarboxylate metabolism. Furthermore, glycine, serine, threonine, glutathione, arginine, and proline metabolism; alanine, aspartate, and glutamate metabolism; and several other metabolic pathways were enriched, including the TCA cycle.

The pathways enriched in hpH were a bit different from npH-enriched pathways, particularly amino acid biosynthesis pathways. In 30 hpH vs 500 hpH, the pathways enriched included galactose and glycerol lipid metabolism and seleno-compound metabolism. The pathways related to amino acid included glycine, serine, and threonine metabolism; alanine, aspartate, and glutamate metabolism; and arginine metabolism, but valine, leucine, and isoleucine metabolism were found only in npH conditions. The TCA cycle was also enriched, but it is less than in npH conditions. The transcriptome analysis of KEGG pathways, which were enriched during this study, also included the majority of the pathways enriched in the metabolome study. To mention the important pathways, they included amino acid metabolism (PATH: ko00260, PATH: ko00400, PATH: ko00730, PATH: ko00900, PATH: ko00270, PATH: ko00280, and PATH: ko00380), carbohydrate metabolism (PATH: ko00630), and selenocompound metabolism (PATH: ko00450).

### qRT-PCR expression analysis of TCA cycle genes

**Fig 10:**
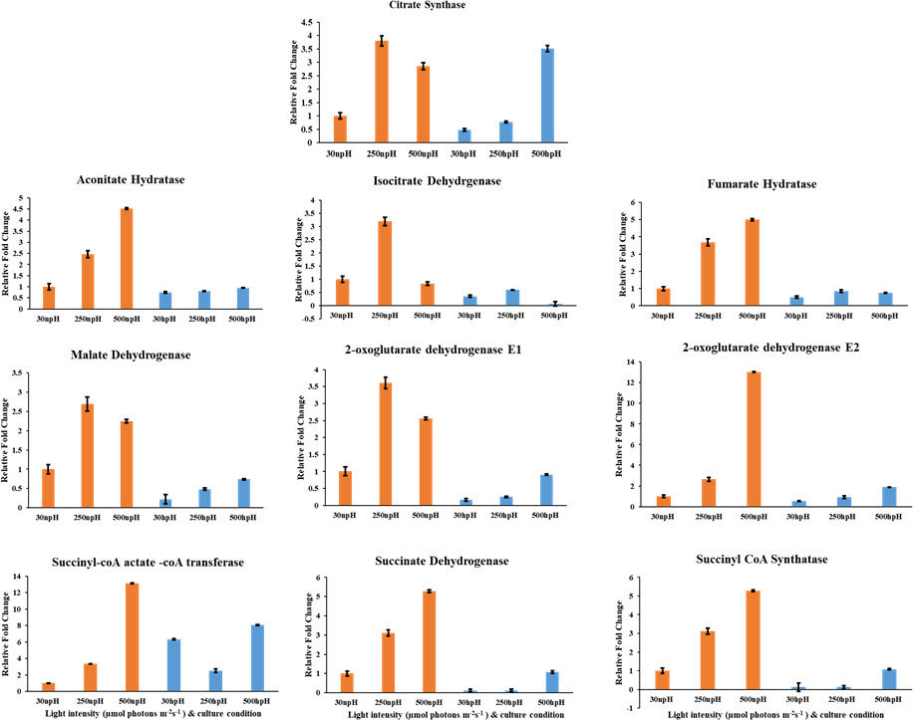
Determination of expression level of Calvin cycle/TCA cycle genes CS, iso-citrate dehydrogenase, AH, FH, MD, OGDE1, OGDE2, SAT, SD, and SS in the npH and hpH conditions at 30, 250, and 500 µmol photons m^−2^s^−1^.

The TCA cycle is also one of the central metabolic pathways of metabolism; the related metabolite expression levels were also enhanced in npH and hpH under high-light conditions. Figure 10 depicts the expression levels of TCA cycle genes from citrate synthase (CS) to malate dehydrogenase (MD). The expression levels of most of the genes in npH and hpH conditions were enhanced with increased light intensity; however, the expression was higher in npH conditions. The expression level of citrate synthase (CS), aconitate hydratase (AH), 2-oxoglutarate dehydrogenase E1 (OGDE1), 2-oxoglutarate dehydrogenase E2 (OGDE2), succinyl-CoA-acetate CoA-transferase (SAT), succinyl CoA synthetase (SS), succinate dehydrogenase (SD), MD, and fumarate hydratase (FH) was relatively high in npH conditions; however, the expression of isocitrate dehydrogenase was slightly reduced in light intensity of 500 µmol photons m^−2^s^−1^. A similar pattern of expression was observed in both npH and hpH conditions (Fig. 10).

## DISCUSSION

*R. alkalitolerans* is one of the versatile photosynthetic bacteria, and it is exposed to fluctuations in light and pH conditions naturally. It becomes imperative to study the transcriptomic and metabolomic responses of the cell. The transcriptome analysis of the *R. alkalitolerans* included the initial mapping of the sequence, and maximum similarity was found with *R. azotoformans*, as it is phylogenetically close to it (12). Bacterial species such as *R. sphaeroides* and *B. viridis* were also considered, as they have been vastly studied for their photosynthetic apparatus energy transfer and metabolism (10, 13, 14). Our recent study shows that cells grown in hpH have a relatively stable photosynthetic apparatus compared to npH-grown cells, along with higher bacteriochlorophyll content and a lower carotenoid to chlorophyll ratio. Furthermore, increased ATP levels and their activity, lower oxidative stress, and increased antiporter expression were observed. Also, studies show that hpH tolerance protects the bacterial photosynthetic apparatus from the detrimental effects of high light and maintains the cell’s homeostatic balance (10). Therefore, the molecular level changes, such as transcriptomic and metabolomic understanding, will provide more insights into the adaptation mechanism in *R. alkalitolerans*.

From the volcano plot analysis, a few of the most upregulated ones were a bifunctional coenzyme of PQQ synthesis protein, PqqA peptide cyclase, and hydrazine synthase subunit β, respectively. Whereas the downregulated genes—formyl tetrahydrofolate deformylase and NADP-reducing hydrogenase subunit HndC—are involved in carbohydrate metabolism by participating in glyoxylate and decarboxylate metabolism. There was another gene coding for methyl malonyl-CoA carboxyltransferase 12S subunit that is involved in leucine, isoleucine, and valine metabolism. This signifies that in npH, the pathways of protein or amino acid and carbohydrate metabolism were upregulated compared to hpH, even at the light intensity of 30 µmol photons m^−2^s^−1^. Similarly, at the high light intensity of the 30 npH vs 500 npH, genes playing a role in porphyrin metabolism were upregulated, which signifies the effect of high light response to decrease in the BChl *a* concentration; these genes were highly expressed. Furthermore, for comparative hpH conditions as well, the upregulated transcripts included the porphyrin pathway-related genes, such as *bchJ* and *bchX*, which were also upregulated, indicating the effect of high light in hpH. Similar studies were also reported earlier for another photosynthetic bacterium (9, 15).

### Core reaction center and light-harvesting antenna complex transcripts overexpress in high light

Overexpression of reaction center and light-harvesting transcripts in npH than hpH signifies that high light impacts the expression of these core reaction center transcripts for their upregulation. The light-harvesting antenna proteins also show that the expression of transcripts encoding the LH1 and LH2, which are *pufA*, *pufB*, *pucA*, and *pucB*, is more in high light intensity. For *pucA* and *pucB*, the fold change was more in hpH than npH, and for LH1, the expression was higher in npH conditions. However, the comparison between the same light intensity in npH and hpH showed a bit of contrast expression for the LH2 transcripts (Fig. 2C). Overall, the majority of the reaction center transcripts were relatively more upregulated in npH conditions than the hpH conditions (Fig. 2C), which indicates relatively lesser turnover in the hpH condition than npH in high light. This could be because of the lesser impact of the highlight in the case of hpH, as evident in the earlier report (10).

Furthermore, a transmembrane protein transcript *pufX*, which is involved in RC-LH1 dimer formation (16, 17), was also found to be more expressed in npH conditions than in hpH high-light conditions (Fig. 2). This is obvious from the objective that in npH conditions, the RC-LH1 dimer content was increased in high-light conditions. In contrast, in the hpH condition, its content was decreased. This observation correlates with the expression of the *pufX* from the transcriptome data. This expression pattern was further validated by the qRT-PCR of all the transcripts for the reaction center, LH1, and LH2 (Fig. 3). The experiment validates that the majority of the reaction center and light-harvesting antenna transcripts were less expressed in hpH than in npH. This exemplifies that cells under npH are under more stress, and to compensate for the loss of protein damage, more expression of transcripts was found.

### BChl *a* and carotenoid biosynthesis pathways are overexpressed

The transcript involved in the biosynthesis pathway of BChl *a* and carotenoid spheroidene/spheroidenone is involved in energy harvesting and excess energy quenching (18). In hpH conditions, relatively higher expression of BChl *a* and carotenoid biosynthesis pathway is found. Furthermore, higher expressions of carotenoids support the conclusion that photosystem protein complexes could be more stable in hpH as these carotenoids have been shown to play an important role in photoprotection and excitation energy transfer (19). Another transcript which is *crtA* coding for the enzyme that converts spheroidene to spheroidenone, its expression was also found to be increased in high-light conditions in both npH and hpH conditions (Fig. 5H). Šlouf et al. (11) reported that the transition between spheroidene and spheroidenone serves as a straightforward and efficient photo-protective strategy, which is likely significant for phototrophic bacteria exposed to light and oxygen.

### Differential effect of highlight on the cell division transcripts in npH and hpH conditions

It also became important to analyze the level of transcripts taking part in cell division, as light intensity has impacted the cell division, cell size, and the generation time (see Fig. S1B at https://github.com/2902-myz/Supplementary-Data-). These upregulated transcripts were found to take part in several cell membrane and peptidoglycan metabolism. The assembly of these proteins occurs in a hierarchical manner, starting with the polymerization of FtsZ at the mid-cell within the cytosol and concluding with the recruitment of enzymes responsible for septal cell wall synthesis, along with their regulatory components, in the periplasm (20). However, two transcripts *murF* and *mraY* were found to be downregulated in the same fashion in high-light conditions in both npH and hpH (Fig. 6A through C), suggesting that they play an important role in peptidoglycan synthesis, which is required during cell division, and this agrees with the previous studies (21–23). However, their lower expression in high light needs to be deciphered. Although another cell membrane transcript, *murG*, is upregulated in npH and hpH conditions under high-light conditions (Fig. 6A through C), it might be compensating for the role of *mraY* and *murF*. Overall, cell division transcripts were more upregulated in high light and npH than in hpH conditions. This leads to an observation that somehow hpH retards the cell division; however, relatively acidic pH leads to more cell division (10). Previous studies show that *Escherichia coli* maintains its cell division under hpH conditions, resulting in elongated cell sizes, in contrast to growth in acidic environments (24).

### Hierarchical metabolite correlation, network analysis, and physiological relevance

The principal component analysis showed a major variation between the sample in hpH compared to npH (see Fig. S4 at https://github.com/2902-myz/Supplementary-Data-) in comparative groups of 30, 250, and 500 µmol photons m^−2^s^−1^ of light intensity, which could be because of metabolic pathways induced by hpH. In a comparative group of 30 npH vs 250 npH, the metabolites that were correlated included BTD, LA, BdA, Put, 11Mo12A, ornithine (Orn), and PdA. BTD stands for 2,3-butanediol; however, it has a range of industrial applications in chemical and cosmetics, but from a physiological perspective, it helps in preventing acidification, regulating intracellular NADH/NAD^+^ balance, and storing carbon as cell growth (25), helping the cells in high light stress conditions. Furthermore, putrescine has been shown to play several important roles in photosynthetic microorganisms. It is an active oxygen scavenger (26), and it promotes the conversion of uroporphyrinogen III to protoporphyrin IX involved in chlorophyll metabolism, especially under stress conditions by maintaining the chlorophyll content (9). Furthermore, 11-methyloctadec-12-enoic acid, a fatty acid, has been identified in many anoxygenic phototrophic bacteria, and the presence of this particular fatty acid becomes a unique characteristic that characterizes this organism. Furthermore, it is important for the structure and function of cellular membranes. It can influence membrane fluidity, which is crucial for the function of membrane-bound proteins, including those involved in photosynthesis (27).

Similarly, Orn plays an important role as a metabolite in Rhodobacter, taking part in the arginase pathway involved in the synthesis of some enzymes such as ornithine 5-aminotransferase and L-ornithine cyclodeaminase along with L-arginine (28). The metabolite pentanedioic acid, a fatty acid, is involved in the Calvin cycle, fatty acid biosynthesis, and tetrapyrrole biosynthesis. It also balances the cellular pyridine nucleotides (NADH/NADPH), which are vital for the cell’s metabolic activities (29). Likewise, many other metabolites were found to be positively correlated with each other, which included Gly, L-5OXP, L-Asp, Phe, OA, and Dp. Gly plays an important role in hydrogen production; L-5OXP, L-Asp, and Phe are involved in glutathione metabolism, which protects against oxidative stress in abiotic stresses in photosynthetic bacteria (30). L-Asp is involved in the aspartate-glutamate pathway, which plays a significant role as a precursor for various amino acids like isoleucine, lysine, methionine, and threonine (31). Apart from that, L-Asp is also part of nucleotide metabolism, TCA cycle, glycolysis, and hormone biosynthesis (31). Similarly, Phe, the precursor of the phenylpropanoid biosynthetic pathway, is an aromatic amino acid that gets activated in high light intensity. This leads to the production of polyphenols, which serve many functions, including the scavenging of reactive oxygen species (ROS) (32). Overall, it can be asserted that these metabolites play important physiological roles to combat stress conditions of high light.

Other metabolites that correlated in these conditions are majorly primary metabolites of amino acids, such as L-Glu, L-Lys, L-Thr, L-Val, L-Ala, 2-KiA, Phy, and OA, which correlated with Dp. These metabolites are involved in all the amino acid metabolic pathways, and 2-KiA is also an intermediate in the catabolism of the branched-chain amino acid leucine. Likewise, Phytol, which is a polyphenol, plays an extensive role in antioxidant production (33), membrane stability (8), and energy dissipation by its derivatives, taking part in non-photochemical quenching in stress conditions (33). The above-mentioned metabolites with an important physiological role in high light increased their interaction more in the high light of 500 µmol photons m^−2^s^−1^ in the comparative pair of 30 npH vs 500 npH (Fig. 7B), which signifies their important physiological role in the metabolism of the cell.

Similarly, for hpH conditions, there were almost the same metabolites interacting, except for a few peculiar metabolites such as L-Pro, β-Ala, and malonic acid (MaA). The role played by these metabolites at high pH is very important. The L-Pro is a well-studied stress-responsive metabolite in plants and photosynthetic algae (34). Under high light, salinity stress, and osmotic stress, proline acts as an osmoprotectant. Salinity stress often accompanies alkaline conditions (35). It has also been shown to play the role of a ROS scavenger, redox balancer, cytosolic pH buffer, and molecular chaperone (34). So, its accumulation in hpH indicates that the hpH condition, along with high light, causes osmotic stress, which leads to L-proline expression but not in npH. Furthermore, β-alanine is a component of pantothenate (vitamin B5), which is integrated into the universal carbon transport molecules Coenzyme A and the acyl carrier protein, present in all living organisms, including plants. Still, it also works as an osmoprotectant in some species and is converted to the antioxidant homoglutathione in others (36). Similarly, MaA is synthesized in bacteria by the malonic acid pathway, which is crucial for phenolic compounds and which plays a proven role in stress responses (37), protecting it in high light.

Furthermore, the majority of metabolites, which had many correlations, also had more interacting partners, along with some other metabolites. In npH, apart from the amino acid and fatty acids, some other metabolites included are ethanolamine (Eta), Nia, 3-hydroxybutyric acid (3HBA), and Cre, which also have interacting partners. Eta is a monoamine and is important in nitrogen metabolism required for bacterial growth (38), whereas Nia is a vitamin B3 family compound involved in enzymatic reactions (39), and 3HBA, which is a monomeric unit for the production of poly-3-hydroxybutyrate. Apart from their physiological importance in high light stress, their interaction increased more at 500 µmol photons m^−2^s^−1^ than at 250 µmol photons m^−2^s^−1^ (Fig. 8A and B). Likewise, in hpH also, the networking among the metabolites increased more at 500 µmol photons m^−2^s^−1^ than at 250 µmol photons m^−2^s^−1^. Some new metabolites also appeared, such as d-Etf, Msa, and Cia, which are sugars and intermediates of the TCA cycle, which is the central metabolic pathway.

### Enriched metabolic pathways, their physiological relevance, and qRT-PCR

Selenocompound metabolism is known for providing membrane stability, enhancing the activity of various antioxidant enzymes in various abiotic stresses in plants (40). So, it might play almost the same role as membrane stability, which is also affected more in npH cells in high light than hpH (Fig. 9). Similarly, pathways—such as valine, leucine, and isoleucine metabolism; glycine, serine, and threonine metabolism; and the TCA cycle—were enriched, which shows that there is a high turnover of amino acids showcasing the impact of high light stress on protein degradation and also intermediates of the TCA cycle, which was enriched in 30 npH vs 500 npH. As photosynthetic membrane proteins show less abundance, it reflects more protein degradation because of stress in pH. However, in hpH conditions, the metabolic pathways that were enriched included the β-alanine metabolism, which is important in stress protection; glyoxylate decarboxylate metabolism; glycine, serine, and threonine metabolism; and lastly, the TCA cycle. This difference in the metabolic pathways in hpH compared to npH indicates a different set of metabolisms operating, providing more protection to the cell in hpH as it also might be posing slight ionic stress/osmotic stress as well. Similarly, lesser TCA cycle enrichment was found in hpH than in npH (Fig. 9C and D).

Furthermore, the qRT-PCR of the TCA cycle, as it is the central metabolic pathway and provides substrate for other pathways, showed that almost all the transcripts showed a relatively lesser expression in hpH than npH. This observation is also in correlation with the pathway enrichment analysis, where the TCA cycle was relatively more enriched in pH high-light conditions than in pH conditions (Fig. 9). The transcriptomics and metabolomic study reveals that several transcripts and metabolites were differently expressed when *R. alkalitolerans* was exposed to high light; however, it was more pronounced in npH.

### Conclusion

The study provides insights into the metabolomics and transcriptomic responses of *R. alkalitolerans* strain JA916^T^ in hpH and npH conditions responding differently to high light stress. It also provides a cross-talk of two different growth conditions, i.e., npH and hpH, with high light influence. The genes encoding the photosynthetic apparatus of *R. alkalitolerans* were impacted by high light in both npH and hpH conditions. However, differential expression of carotenoids and Bchl *a* synthesizing genes was relatively more expressed in hpH, emphasizing more energy trapping, more photo-protection, and lesser impact on the photosystem transcripts. Similarly, the metabolomics responses indicated that several metabolic pathways were enriched, including amino acid biosynthesis and degradation pathways, along with the TCA cycle, which were more enriched, compensating for the impact of the high light stress. Several metabolites playing an essential role in photoprotection (phytol, L-proline, and putrescine), chlorophyll metabolism (L-aspartic acid and putrescine), and amino acid metabolism (L-alanine, valine, threonine, etc.) were found to have more correlation and network interaction among them. Some peculiar metabolites in hpH were also found, implying their role in high light and hpH (L-proline). Overall, the transcriptomic and metabolic responses of *R. alkalitolerans* were not only in response to different conditions like npH, hpH, and also in combination with high-light conditions. These findings show that under diverse conditions, the photosynthetic bacteria *R. alkalitolerans* have developed an effective photo-protective mechanism that replenishes the detrimental effects of high light stress.

## MATERIALS AND METHODS

### Bacterial cell culture and cell harvest

Bacterial cultures were grown in Biebl and Pfennig’s medium with sodium pyruvate (3 g/L) as carbon source and ammonium chloride as nitrogen source (0.4 g/L) in light/anaerobically at 25°C in a glass bottle with glass cork stoppers to avoid air infiltration as described previously (12, 16). *R. alklitolerans* strain JA916^T^ culture was grown in three light intensities of 28−30 µmol photons m^−2^s^−1^ (optimum light), 250−255 µmol photons m^−2^s^−1^, and 500 ± 5 µmol photons m^−2^s^−1^ under two pH conditions pH 6.80 ± 0.05 (npH) and pH 8.60 ± 0.05 (hpH) (10). The cultures were maintained by inoculating 1.125 mL of inoculum culture in 300 mL of Biebl and Pfennig’s media prepared in 25 mM of Tris buffer. Cells were harvested at late log phase (approximately at OD 1.4−1.5 at 660 nm) (41) by centrifuging at 15,000 × *g* for 20 min, washed with 20 mM HEPES pH 7.5, and stored at −80°C.

### Total RNA extraction, cDNA synthesis, and quantitative real-time PCR

Total RNA was extracted using STRN50-1KTspectrum TM Plant total RNA Kit according to the manufacturer’s protocol. RNA concentration was calculated at 260 nm with a NanoDrop 2000 Spectrophotometer (Thermo Scientific, USA). cDNA was synthesized using a cDNA synthesis kit (TAKARA) by following these conditions: priming at 65°C for 15 min and reverse transcription at 42°C for 1 h in a 20 µL reaction volume. The RNA extracted was also used for whole transcriptome analysis.

Forward and reverse primers were designed for pathway target genes, including carotenoid spheroidene, BChl *a*, reaction center genes PHB biosynthesis, TCA cycle, and cell division, based on the available gene sequences of *R. azotoformans KA 25* (Ensembl bacteria). Using *R. alkalitolerans* cDNA, genes were amplified, and the 2^−ΔΔCT^ method (the Applied Biosystems User Bulletin No. 2 [P/N 4303859]) (42) was used to measure expression levels of target gene and housekeeping genes (recA). RT-PCR was carried out in Eppendorf Mx3000P (Germany) multiplex quantitative PCR system using SYBR Green PCR master mix (Kappa, South Africa). Target genes were amplified using the following program: one cycle at 95°C for 2 min, followed by 40 cycles of 30 s at 95°C for template denaturation, 30 s at 60°C, and 20 s at 72°C, followed by the dissociation (melting) curve. Each reaction was carried out in triplicate with 50 ng of cDNA as template. Templates (cDNAs) were used from three biological replicates, where cDNAs from three individual plants were pooled for each biological replicate. The relative fold difference was determined using the 2^−ΔΔCT^ formula (43). We checked the efficiency of the target and reference as per the Applied Biosystems User Bulletin No. 2 (P/N 4303859) to ensure that both genes amplify with approximately equal efficiency.

### Transcriptome analysis

Whole transcriptome sequencing was performed using Illumina (Novaseq 6000), 150 P platform. The raw reads obtained were analyzed. The raw data quality was checked using Fast QC and Multi QC (44) software. The data were checked for base call quality distribution, % bases above Q30, %GC, and sequencing adapter contamination. All the samples have passed the QC threshold (Q30 > 85%). The raw sequence reads were processed to remove adapter sequences and low-quality bases using fastp v0.12.4 (45) with default parameters. Reads shorter than 50 bp were removed from further analysis. From the quality-trimmed data, rRNA reads were removed using bbmap’s v38.18 bbduk algorithm.

### Read alignment

The pre-processed reads were mapped onto an indexed *R. azotoformans str. KA25* (GCA_003050905) reference genome using Bowtie2 v2.4.5 aligner (46). On average, 43.40% of the reads aligned onto the reference genome.

### Expression profiling

The level of gene expression values was fetched out utilizing *R. azotoformans str. KA25* (GCA_003050905) as a reference species for identification and read counts using feature Counts v2.0.0 software (47).

The expression similarity between the samples and biological replicates was analyzed by PCA and Spearman rank correlation (Correlation_matrix.xlsx) using the normalized values of expressed genes. Spearman’s rank correlation, which is a non-parametric test, was employed to measure the degree of association between two variables.

### Differential expression analysis

The samples were categorized into two groups for differential expression analysis: control and treated groups. Differential expression analysis was performed utilizing the DESeq2 v1.34.1 package following the normalization of read counts to variance-stabilized normalized counts. Genes that had fewer than five reads in any of the samples were excluded from the analysis.

Genes with absolute log2 fold change ≥1 and adjusted *P*-value ≤ 0.05 were considered significant. The expression profile of differentially expressed genes among the samples was illustrated through volcano plots and heatmaps (48). A volcano plot was made to show the differential expression profiles of genes.

### Gene ontology and KEGG analysis

Differentially expressed genes were used for Gene Ontology (GO) and KEGG pathway enrichment analysis. GO information was retrieved from the Uniprot database. The KO IDs were obtained from the KAAS (49) server, taking the protein file of the organism as input. Enrichment analysis for GO and KEGG pathways was performed using the Cluster Profiler v4.2.2 (50). The Cluster Profiler package provides an enricher (enrichment analyzer) function for hypergeometric tests. GO and KEGG were considered significant with an adjusted *P*-value of ≤0.05, resulting from multiple tests.

### Metabolite extraction and gas chromatography–mass spectrometry analysis

A lyophilized cell powder sample weighing about 20−25 mg was subjected to extraction using 480 µL of pure methanol, to which 20 µL of 0.2 mg mL^−1^ ribitol (adonitol) solution was added as an internal standard. The mixture was shaken vigorously for 2 min and subsequently heated to 70°C for 15 min. An equal volume of water was added and shaken vigorously again, 250 µL of chloroform was added, and the mixture was thoroughly mixed. This mixture was subjected to centrifugation at 2,200 × *g* for 10 min at room temperature, which was approximately 25°C. The supernatant was removed and evaporated using a speed vacuum rotator at 35°C.

The dried residue was subsequently dissolved in 40 µL of a 20 mg/mL solution of methoxamine hydrochloride in pyridine and incubated for 90 min at 37°C. A total of 60 µL of MSTFA (N-methyl-N-(trimethylsilyl) trifluoroacetamide) was added, and the whole mixture was incubated for 30 min at 37°C. Following the derivatization process, the sample was placed into a gas chromatography–mass spectrometry (GC-MS) vial that contained an insert. The injection volume was set at 0.2 µL, utilizing the Split Mode for injections, with a split ratio of 5 (51).

GC-MS analysis for untargeted analysis consisted of a Shimadzu Gas Chromatogram (GC-2010 plus) coupled with a mass spectrometer (TQ 8050) and an auto sampler (AOC-20s)–auto injector (AOC-20i). Analysis was conducted using SH-Rxi-5Sil MS capillary column (30 m × 0.25 µm, 0.25 mm; Restek Corporation, USA) and helium with a flow rate of 1 mL min^−1^ as carrier gas. The procedure involves isothermal heating at 80°C for 2 min, followed by a temperature increase at a rate of 5°C per minute until reaching 250°C. This is then maintained for 2 min, followed by a final ramp-up at 10°C per minute, with a hold time of 24 min. The overall duration for the GC-MS analysis was 67 min, which included a solvent delay of 4.5 min. The integration of the chromatogram and the analysis of mass spectra were performed using Shimadzu Lab Solutions software (GC-MS Solution Version 4.53SP1), and the NIST17s spectral library was used for derivatized metabolite identification. Obtained metabolites were analyzed by Metaboanalyst software (V.6.0). Interaction and correlation analysis between the metabolites was analyzed by using the R program and Cytoscape software.

## Data Availability

The sequence reads can be obtained from the National Center for Biotechnology Information (NCBI) using the accession number PRJNA1297354. The supplementary data are available at the following link: https://github.com/2902-myz/Supplementary-Data-.
